# ‘People like me don’t get support’: Autistic adults’ experiences of support and treatment for mental health difficulties, self-injury and suicidality

**DOI:** 10.1177/1362361318816053

**Published:** 2018-11-29

**Authors:** Louise Camm-Crosbie, Louise Bradley, Rebecca Shaw, Simon Baron-Cohen, Sarah Cassidy

**Affiliations:** 1 Coventry University, UK; 2 Coventry and Warwickshire Partnership NHS Trust, UK; 3 University of Cambridge, UK; 4 Cambridgeshire and Peterborough NHS Foundation Trust, UK; 5 University of Nottingham, UK

**Keywords:** Autism spectrum condition, mental health, self-injury, suicide, support, treatment

## Abstract

Autistic people are at high risk of mental health problems, self-injury and suicidality. However, no studies have explored autistic peoples’ experiences of treatment and support for these difficulties. In partnership with a steering group of autistic adults, an online survey was developed to explore these individuals’ experiences of treatment and support for mental health problems, self-injury and suicidality for the first time. A total of 200 autistic adults (122 females, 77 males and 1 unreported) aged 18–67 (mean = 38.9 years, standard deviation = 11.5), without co-occurring intellectual disability, completed the online survey. Thematic analysis of open-ended questions resulted in an overarching theme that individually tailored treatment and support was both beneficial and desirable, which consisted of three underlying themes: (1) difficulties in accessing treatment and support; (2) lack of understanding and knowledge of autistic people with co-occurring mental health difficulties and (3) appropriate treatment and support, or lack of, impacted autistic people’s well-being and likelihood of seeing suicide as their future. Findings demonstrate an urgent need for autism treatment pathways in mental health services.

## Introduction

Adults diagnosed with autism spectrum conditions, (ASC, also commonly referred to as autism spectrum disorder (ASD)), are at high risk of co-occurring mental health conditions (Lever and Geurts, 2016; Wigham et al., 2017), non-suicidal self-injury (NSSI; Maddox et al., 2017), and suicidality (Cassidy et al., 2014a, 2018a; Hedley and Uljarević, 2018; Hirvikoski et al., 2016). However, mental health, self-injury and suicidality in autism are poorly understood and under-researched (Cassidy and Rodgers, 2017), with a shortage of professionals trained in both autism and mental health (Raja, 2014), lack of appropriate assessment tools (Cassidy et al., 2018b, 2018c) and therapies (Ghaziuddin et al., 2002) for this group. Given that autistic adults without intellectual disability (ID) are at highest risk of contemplating suicide (66%, Cassidy et al., 2014a), and dying by suicide (Hirvikoski et al., 2016), the current study focuses on autistic adults without co-occurring ID (henceforth autistic adults^1^).

Lack of support for autistic adults has been associated with increased risk of depression and suicidality (Cassidy et al., 2018a; Hedley et al., 2017). Autistic adults also report a significantly higher number of unmet support needs than the general population (Cassidy et al., 2018a); autistic adults and children struggle to obtain appropriate support post diagnosis (Crane et al., 2016; Jones et al., 2014); and autistic young adults (16–25 years) face challenges in accessing appropriate treatment for mental health problems (Crane et al., 2018). Hence, despite autistic people being at significantly increased risk of mental health problems and suicidality than the general population, they appear to be less likely to be able to access support and treatment for these difficulties.

Reasons for this lack of treatment and support are likely complex. First, mental health professionals and psychiatrists are generally not trained in recognising and understanding ASC, meaning services are unprepared to adapt support and treatment to this group (Raja, 2014). Second, there are a lack of appropriate validated assessments to effectively identify mental health problems such as depression (Cassidy et al., 2018b) and suicidality in autistic adults (Cassidy et al., 2018c; Hedley et al., 2018). Third, there is a lack of appropriate adapted psychological therapies for autistic people (Ghaziuddin et al., 2002). These challenges are likely greater for autistic adults without ID (commonly referred to as Asperger Syndrome in ICD-10, but subsumed into ‘autism spectrum disorder’ in *Diagnostic and Statistical Manual of Mental Disorders* (5th ed.;DSM-5; American Psychiatric Association (APA), 2013)), who compared to other autism subtypes experience greater delay in diagnosis (Howlin and Asgharian, 1999) and accessing treatment (Bowker et al., 2011), increased risk of being initially misdiagnosed with a mental health problem (Au-Yeung et al. in press; Dossetor, 2007; Punshon et al., 2009) and lack of post-diagnostic support (Howlin, 2008; Jones et al., 2014). Therefore, autistic adults without ID are likely encountering professionals without the necessary skills or tools to effectively assess, support or treat autistic adults experiencing these difficulties.

Little is known about autistic adults’ experiences of receiving treatment and support for mental health problems, self-injury and suicidality, with a majority of studies being quantitative. Autistic people feel their voices have been excluded from research (Milton and Bracher, 2013), so a participatory approach is advocated in research (Fletcher-Watson et al., 2018; Pellicano et al., 2014). Hence, this study explored autistic adults’ experiences of treatment and support for mental health problems, self-injury and suicidality from their own perspective, using a participatory approach – autistic adults helped define the aims, design and questions used in the research. We explore barriers and enablers for autistic adults when accessing support and treatment, to make recommendations for future research, policy and practice to improve access to mental health services for autistic adults without ID.

Given the lack of previous research, we use an exploratory qualitative approach, with broad research question, to capture sufficient breadth and depth of participants’ experiences (Braun and Clarke, 2013). Open-ended questions in qualitative surveys allow access to a larger sample than other qualitative methods (Robson, 1993). These benefits are magnified when the research question is sensitive, as online surveys provide privacy and anonymity, extending the potential reach further (Braun et al., 2013). Open-ended online questions also allow an investigation into participants’ understanding of their own experiences without researcher, or other participants, involvement that may bias their responses (Robson, 1993). When similar experiences are reported, it provides compelling evidence for a shared understanding of the issues being explored. As many autistic people have communication and social interaction difficulties (APA, 2013), they may find interviews or focus groups challenging or uncomfortable. Hence, the use of an online survey allows a wider range of autistic people to participate.

## Method

### Participants

A total of 200 autistic adults were included in the qualitative analysis (122 females, 77 males and 1 unreported), aged 18–67 (M = 38.9 years, SD = 11.5). The age of autism diagnosis ranged from 2 to 59 years (M = 34.1 years, SD = 13.8). A majority (90.4%) of participants had a mental health diagnosis, most prevalent being depression and anxiety. The most prevalent autism subtype was Asperger syndrome (Table 1). In relation to treatment for mental health, self-injury and suicidality (n = 197), 164 participants (83.2%) were currently receiving/had previously received treatment, 29 participants (14.7%) needed/currently needed treatment but had not received it and 4 participants (2%) did not need treatment (Table 2). In relation to support, (n = 198) 164 participants (82.8%) reported that they would like to have support or currently receive support. Of those who reported they would like to receive support and answered the question on types of support received (n = 159), 76 participants (47.8%) currently received none (Table 3).

**Table 1.** Participant demographics.

Mental health and other diagnoses	Men (n = 67)	Women (n = 112)	Total (n = 179)
Depression	60 (89.6%)	99 (88.4%)	159 (88.8%)
Anxiety	51 (76.1%)	91 (81.3%)	142 (79.3%)
Other	13 (19.4%)	27 (24.1%)	40 (22.3%)
OCD	11 (16.4%)	28 (25.0%)	39 (21.8%)
Personality disorder	4 (6.0%)	20 (17.9%)	24 (13.4%)
Bipolar	4 (6.0%)	11 (9.8%)	15 (8.4%)
Anorexia	1 (1.5%)	12 (10.7%)	13 (7.3%)
ME/CFS	3 (4.5%)	10 (8.9%)	13 (7.3%)
Epilepsy	2 (3.0%)	5 (4.5%)	7 (3.9%)
Schizophrenia	2 (3.0%)	4 (3.6%)	6 (3.4%)
Tourette’s	2 (3.0%)	2 (1.8%)	4 (2.2%)
Bulimia	0 (0.0%)	4 (3.6%)	4 (2.2%)
ASC subtype	Men (n = 77)	Women (n = 121)	Total (n = 198)
AS/HFA	61 (75.3%)	104 (80.8%)	165 (78.7%)
Autism/classic autism/ASC	9 (14.3%)	11 (18.3%)	20 (16.8%)
PDD/PDD-NOS	0 (10.4%)	1 (14.2%)	1 (12.7%)
Other	7 (10.4%)	5 (4.2%)	12 (6.6%)

OCD: Obsessive Compulsive Disorder; ME: Myalgic Encephalopathy; CFS: Chronic Fatigue Syndrome; AS: Asperger syndrome; HFA: high functioning autism; ASC: autism spectrum condition, PDD-NOS: pervasive developmental disorder, not otherwise specified; PDD: pervasive developmental disorder; other: no specific ASC subtype specified.

**Table 2.** Comparison of treatments participants received or were currently receiving to treatments participants needed but had not received, stratified by sex.

Treatments	Treatments previously received/currently receiving			Treatments previously/currently needed but not received		
	Male (n = 65)	Female (n = 99)	Total (n = 164)	Male (n = 8)	Female (n = 21)	Total (n = 29)
Mental health	56 (87.5%)	91 (93.8%)	147 (91.3%)	6 (75.0%)	17 (81.0%)	23 (79.3%)
Self-injury	7 (10.9%)	20 (20.6%)	27 (16.8%)	0 (0.0%)	7 (33.3%)	7 (24.1%)
Thoughts of ending life	17 (26.6%)	38 (39.2%)	55 (34.2%)	5 (62.5%)	8 (38.1%)	13 (44.8%)
Other	13 (20.3%)	18 (18.6%)	31 (19.3%)	3 (37.5%)	3 (14.3%)	6 (20.7%)

**Table 3.** Types of support received and would ideally have liked, stratified by sex.

Support	Currently receive support			Would ideally like support		
	Male (n = 60)	Female (n = 99)	Total (n = 159)	Male (n = 61)	Female (n = 101)	Total (n = 162)
Mental health care	13 (21.7%)	22 (22.2%)	35 (22.0%)	33 (54.1%)	66 (65.3%)	99 (61.1%)
Mentoring	9 (15.0%)	10 (10.1%)	19 (11.9%)	33 (54.1%)	101 (67.3%)	101 (62.3%)
Social activities	5 (8.3%)	7 (7.1%)	12 (7.5%)	29 (47.5%)	53 (52.5%)	82 (50.6%)
In the home	7 (11.7%)	10 (10.1%)	17 (10.7%)	15 (24.6%)	31 (30.7%)	46 (28.4%)
Employment	5 (8.3%)	6 (6.1%)	11 (6.9%)	33 (54.1%)	40 (39.6%)	73 (45.1%)
Health care	6 (10.0%)	8 (8.1%)	14 (8.8%)	16 (26.2%)	30 (29.7%)	46 (28.4%)
Finance	5 (8.3%)	6 (6.1%)	11 (6.9%)	20 (32.8%)	40 (39.6%)	60 (37.0%)
In the community	4 (6.7%)	8 (8.1%)	12 (7.5%)	15 (24.6%)	25 (24.8%)	40 (24.7%)
Organisation	1 (1.7%)	4 (4.0%)	5 (3.1%)	16 (26.2%)	50 (49.5%)	66 (40.7%)
Education	5 (8.3%)	13 (13.1%)	18 (11.3%)	19 (31.1%)	39 (38.6%)	58 (35.8%)
Other	5 (8.3%)	14 (14.1%)	19 (11.9%)	17 (27.9%)	13 (12.9%)	30 (18.5%)

Participants’ data were drawn from the mental health in autism survey, a large online survey developed with the Coventry Autism Steering Group, which explored mental health problems, suicidality and thoughts of ending life in autistic adults. Participants were recruited to this survey from the United Kingdom through the Cambridge Autism Research Database (CARD), charities and support groups, educational institutions and social media.

### Materials

A survey exploring mental health, self-injury and suicidality in autistic adults was developed in partnership with an autistic steering group across six focus groups, facilitated by researchers. S.C., L.B. and R.S. Full details of the dataset and survey questions are available in a previously published study (Cassidy et al., 2018a). The steering group consisted of eight autistic adults without ID (six female, two male), who consented to share their experiences of mental health problems, self-injury or suicidality to inform future research and practice. The researchers designed a questionnaire to capture the discussion of these experiences. The steering group fed back on three drafts of the survey to ensure questions were comprehensive, relevant and clear to autistic people.

Data from the survey concerning experiences of support and treatment are presented here. Participants completed a self-report questionnaire, exploring experiences of treatment and support using closed and open-ended questions. Closed questions asked whether participants had ever needed or received treatment or support, what they currently/previously needed treatment for (mental health problems, self-injury, thoughts of ending life or other), the areas which they currently/have received support in and the areas they would ideally like support. Subsequently, open questions asked: ‘Please describe your experience of trying to get treatment for your condition(s). (*For example, what has your experience been like, and how could it be improved? Are there any barriers preventing your access to treatment?*)’ and ‘If you wish, please provide more details of your experiences of support. (*For example, do you currently get the support you need? Does anything make it difficult to get the support you need? Is any support you are currently receiving/have received appropriate for your needs? Could anything be improved? If so, how could it be improved?*).’

If participants indicated that they would like support, but do not currently receive any, they were asked: ‘If you wish, please describe how has this affected you?’. If participants indicated that they currently/have received support, they were asked: (1) ‘What have been the positive consequences of this support (if any)?’ and (2) ‘What have been the negative consequences of this support (if any)?’.

### Ethics

All elements of the survey were designed to minimise potential distress. Participants were given full details about the nature of the research and contact details of relevant support organisations before, during and after the survey. It was made clear to participants that no questions were compulsory. The ethical approach was discussed with the steering group to ensure it was acceptable to autistic people. This study was granted ethical approval by the Coventry University Psychology Ethics Board, and the CARD Advisory board, prior to contacting participants registered in CARD.

### Data analysis

Thematic analysis was used to analyse data from the open-ended survey questions (Braun and Clarke, 2006). Analysis was carried out by L.C.-C., who was not involved in the steering group discussions facilitated by S.C., L.B. and R.S., to avoid unnecessary bias. However, as thematic analysis is an active process, the researchers acknowledge that their own experiences and preconceptions as non-autistic people naturally influenced the themes to an extent. L.C.-C. undertook the thematic analysis from an essentialist theoretical position with experiences understood as the participant’s reality. Analysis was data driven and carried out inductively, themes were derived from the data, as opposed to from a pre-existing theoretical framework or previous research (Potter and Wetherell, 1987). L.C.-C. discussed themes with S.C. and L.B., who offered feedback and helped refine themes to ensure interpretation of the data was valid.

## Results

The overarching theme on which this article focuses was that tailored treatment and support is both beneficial and desirable. This theme was dominant throughout participants’ experiences, for example, ‘it has become very apparent that approaches to treatment have to be tailored to patients with an ASC’ (78F)^2^. Underneath this overarching theme, sat three themes: ‘people like me don’t get support’, ‘lack of understanding and knowledge’ and ‘well-being’ (Table 4).

**Table 4.** Thematic table showing the overarching theme, three themes and eight sub-themes.

Tailored support is beneficial and desirable							
‘People like me don’t get support’			Lack of understanding and knowledge			Well-being	
Dismissed for treatment or support because seen as ‘coping’	Support geared towards children	Long waiting lists and lack of funding	Obstacles to accessing and receiving treatment and support	Not believed or listened to	Not suited to my needs	Negative impacts	Positive and enabling

Treatment and support was not distinguished in the analysis because participants used them interchangeably. Frequency count of themes and sub-themes are reported, not as a measure of prevalence, but given the large sample of responses to help gauge the extent of shared experience (Braun and Clarke, 2013).

## ‘People like me don’t get support’

This theme explores participants’ experiences of difficulty finding appropriate treatment and support, as autistic adults with mental health difficulties:

I haven’t requested any, because people like me don’t get support. Any time I have enquired about such things that has been made very clear. At this point I don’t even know who I might request help from. (654M)

For many participants, this lack of treatment or support was explicitly linked to their co-occurring autism and mental health diagnoses (16 participants), which made them ‘too complicated’ (36F), and ineligible for services: ‘if you have [ASD] then mental health say it’s not their problem’ (157M); and ‘unless you have a co-morbid learning disability^3^, you’re on [your] own’ (237F).

For some then, the experience of being autistic and having co-occurring mental health issues seemed one of falling through a gap in available services (37 participants).

### Dismissed for treatment or support because viewed as ‘coping’

This theme refers to ‘coping’ in the broadest sense, rather than ‘with’ a particular singular thing. Participants described how their need for support was dismissed due to non-autistic people’s (mis)perceptions of ‘high-functioning’ ASC (26 participants). Specifically, autistic people were viewed as ‘coping’ (474F), ‘functioning’ (56F) or ‘managing’ (28F) even when they were not because they were too ‘high-functioning’, independent, in employment or at University, which resulted in them being excluded from relevant treatment and support:

I am high-functioning and an adult … I feel ‘lost’…. I am too high functioning for most ASD programming in my area, but not neurotypical enough to function well in conventional work and social situations and environments. (650F)

However, being in employment, for example, did not necessarily mean that the person was coping and did not need support or treatment:

I think some of the issues with understanding is that some people don’t think you can be really struggling if you are managing to go to work/uni etc. Of course in hindsight with the Asperger’s diagnosis that is just partly because dropping the routine would be even worse. I was also conscious that I was safer not in the house. (235F)

### Support geared towards autistic children

Participants identified that support is tailored towards children and their parents (nine participants):

[support] in past – good especially at autism specific college. i rarely needed to explain autism or myself … now not good at all – life is hard and people seem to fighting [against] me who should be there to help. (153F)

### Long waiting lists and lack of funding

Participants identified long waiting lists and lack of funding (from the National Health Service (NHS) in the United Kingdom; 37 participants) as reasons for there being no treatment or support available to them, even if actively suicidal (9 participants):

I tried to kill myself and I was really f***ed up and needed counselling. The NHS waiting list was nine months. You [can’t] wait nine months when you’re suicidal … I only got through that period by abusing drugs. (272F)

Many participants gained access to the treatment and support they needed by paying for it themselves, while those who could not afford to went without (37 participants):

if I had not been able to borrow the money to see one privately, I would still be living a nightmare full of untreated anxiety and self-hatred because I thought it was my fault I was different … (11F)

Participants also cited a lack of available treatment in their local area (12 participants), due to their refusal ‘to fund anything’ (744F). Location acted as a barrier for those ‘unable to access treatment that involves travel as I lack energy and cannot use buses’ (131F), or more generally because they simply ‘can’t get there and back again’ (192F).

## Lack of understanding and knowledge

Experiences of professionals’ ‘very poor knowledge of autism’ (181F) played a key role in many negative experiences (89 participants):

The biggest difficulty in getting the support I need is the lack of understanding of autism. Even after decades of research, many institutions still don’t have the first clue in dealing with such a condition, and hence I have found only select places deal with autism specifically. The support I have received I feel isn’t suitable enough. No ordinary counselor can understand autism, and the social aspects provided by a support worker haven’t helped me to end my feelings of rejection … If there was someone who both understood the condition and me, I feel progress would be made. (750M)

Many participants felt a need for professionals to be better trained in autism to ‘realise it may not always be possible to accurately read a person with autism’ (120F). Participants felt that this up-skilling of autism knowledge was not their responsibility: ‘some of them have asked to borrow books from me about autism or asked me to teach them about it and I feel that shouldn’t be down to me’ (181F). These experiences suggest that professionals were often ‘well-meaning’ (176M) but lacked knowledge of the different way in which autistic people communicate and socially interact, and the implications for their treatment and support.

The following sub-themes will continue to address how professionals lack of understanding of mental health in autism acted as a barrier to accessing appropriate treatment and support.

### Obstacles to accessing and receiving treatment and support

The road to obtaining treatment and support for many autistic people was littered with obstacles, from the initial self-acknowledgement that treatment and support was needed, to the point of receiving it (34 participants):

I recognise that I often don’t realise just how bad things have become. In the last year I have started thinking about suicide, even though I don’t want to die, and that has been the thing that’s made me realise how bad things might be. (375F)

Subsequently, the process of asking for treatment and support lacked transparency and felt daunting and complex: ‘I don’t know how or what I would say or how I would explain or then what would happen’ (298F). Asking for support in itself required a specific skill set: ‘how does someone who need support self-advocate for support because that’s what they ask for’ (47M), and for some, the challenges outweighed the potential benefits: ‘I have not requested it – I find it very difficult seeking help’ (165M).

After successfully obtaining treatment or support, communication difficulties mounted once face to face with a professional:

I felt like I was having a breakdown inside but I didn’t know how to make the inside feelings show to other people. (375F)

Communication difficulties were exacerbated further when ‘in crisis’ and in an unfamiliar and noisy environment such as a hospital:

I can’t always communicate if I’m in crisis. Ward rounds in the hospital would always be awfully stressful for me, having to talk in front of a room of people. So are meetings with my Psychiatrist. I feel comfortable with my care coordinator now and she’s learnt to do things like ask me direct questions rather than open ones, but not everyone is so accommodating. (133F)

### Not believed or listened to

When seeking professional help for mental health problems, many participants felt their symptoms were discarded (29 participants):

With hindsight a massive barrier was that I just don’t show emotion the way other people do so aside from when I was very anorexic no one believed there was anything wrong with me or that I needed help. There are specific things depressed people are supposed to do, but I don’t do them. (18F)

Other participants described being ‘not believe[d] … and told … to go away’ (66F). These experiences unsurprisingly led to ‘loss of trust’ (734M) towards professionals and services for some (19 participants). However, not all experiences were negative. For example, one participant used contrasting experiences to demonstrate the power of being listened to and not being pre-judged:

I have had my share of ineffectual medications and therapies, including ignorant psychiatrists and counsellors who made things worse rather than better because they seemed to decide what the problem was and what was best without actually listening to me. However, I have also had some very good counsellors and GPs who have listened, been good at talking to me in a way I can engage with, and worked with me rather than simply talking at me. (435M)

### Not suited to my needs

Continuity of care was highly valued (12 participants): ‘going back to the same person was good because it takes me ages to form a good rapport’ (158F). Appointments, however, were not long or frequent enough to ‘make yourself well’ (47M) and tainted by worries of them coming to an end (eight participants):

NHS counselling is good for people who can immediately connect to another person. If you have autism it takes time, and by the time you are starting to make some kind of connection you have run out of sessions. (120F)

I guess I’m quite dependent upon the support now, and when I am better enough to be discharged from mental health services where I receive most of my support, I will feel very ‘alone’. (133F)

Lack of understanding of autism also played out in experiences of treatment and support that were not necessarily fit for purpose (38 participants), such as receiving cognitive behavioural therapy (CBT) for mental health problems. Experiences were not homogeneous. Some participants argued CBT was an effective treatment (eight participants):

CBT was more effective than I had thought it would be. It explained my thought processes and why I behaved the way I did. (136F)

However, many argued it was not (22 participants), particularly if it was not adapted to their needs:

I had communication issues with each therapist because they expected me to be neurotypical, so I would take things too literally and they thought it was a defense mechanism, or I’d try to explain meltdowns and they focused on my thoughts rather than how to deal with over-reactive sensory perception. (4F)

## Well-being

Although the survey questions were phrased to explore treatment and support in relation to mental health, self-injury and suicidality, interestingly participants’ responses frequently referenced their overall well-being. This theme explores those experiences of treatment and support, which when negative adversely impacted the participant’s well-being, while in contrast when positive enhanced their well-being.

### Negative Impacts

Experiences of perceived inappropriate support made participants feel worse about themselves and their (dis)abilities, to the extent that it was actually disempowering (14 participants):

Having a mencap support worker when I am not learning disabled has actually made me feel worse. I feel like I’m falling into a pit of incapacity. (4F)

For other participants, the absence of appropriate treatment and support was at the root of the negative impact on their lives (51 participants):

I used to have a lot of support and since the cuts have really struggled – I’m forgetting meds, have lost lots of skills and confidence and am stuck indoors now all the time, I am ill but my mum is old now so can’t take me to gps and when we go they do not listen as they dont understand me … support workers used to help me do all the times I just ticked but I lost them – now I get sore and infections because I forget to wash and struggle to find energy, I used to volunteer and also do courses – now I do nothing- im alone almost all the time … I’ve got to the point where I wonder what the point of me being here is – I dont contribute anything … I have no life and no purpose … (734M)

In the absence of appropriate treatment and support, many participants were reliant on informal support from family and friends (19 participants). Feeling conflicted about this support was not unusual. Participants often felt entirely dependent on this support, as external support was inadequate in addressing their needs, but wanted to become more independent and felt ‘guilty’ (7F). Participants wrote of the burden they felt on family and friends: ‘I would like to feel that I was worth some support instead of what I feel at the moment which is that I’m a useless scrounging burden on the people I care about’ (32F), some wrote of not reaching out for help in case they were *seen* as a burden:

I don’t have many people to turn to when I feel low, so most of the time, I just toss and turn with waking nightmares and don’t tell anyone, because I don’t want to wear out the goodwill of the friends I’ve got. (118F)

Participants also wrote of the toll that supporting them was having on their family and friends, for example, the ‘completely unpaid’ friend who was ‘seriously damaging their health’ (75F). Another participant wrote,

The support doesn’t really meet my needs and so my mother has to do more for me and her health hasn’t been too good really. so sometimes I try to suffer in silence so that I do not let her in my flat so [that] she cannot see what a mess I am in. and that I haven’t food in to eat. (700M)

Participants also felt excluded from society and the local community: ‘I miss out on my vibrant town’s many events because I can’t cope with the noise and crowds on my own’ (131F). Another wrote of participating ‘in the world through an ever-smaller keyhole’ (172F) in reference to activities she no longer felt able to complete, such as going to the shops. Another participant’s life was ‘very limited’ and she was not ‘fulfilling’ her ‘potential’ in that she wanted to ‘contribute to the community’ but did not ‘really know how’ (587F). Other participants experienced exclusion from society in the wider sense. For example, ‘for people with an ASC it is likely that they will be rejected by society because we don’t fit in … The idea of what normal … is shrinking [and] more and more people are being excluded from society’ (34F).

Absent or inappropriate support caused participants to feel hopeless, isolated and alone (eight participants): ‘I am stuck struggling on my own and feeling very hopeless that my situation will ever change for the better’ (32F); and ‘I have great difficulty avoiding hopelessness and depression and do not know when to reach out for help’ (149F). In relation to isolation, participants wrote of feeling ‘alone with my problems’ (197M) and feeling ‘desperate and isolated because I don’t receive any support’ (375F).

Thoughts of ending life were a reality for some participants, who described suicide as an inevitability (four participants):

My sense is that support of any kind just won’t change anything in a practical way. In some ways I feel I’m using the therapy as palliative care until I have enough energy and commitment to actually die. (646M)

### Positive and enabling

In stark contrast, many participants’ experience of receiving treatment and support was positive and directly improved aspects of their mental health and well-being (63 participants). Participants wrote of overcoming a range of specific mental health problems (10 participants), from ‘phobias and anxieties’ (54F) to ‘I now have my OCD under control, I am totally cured of anorexia’ (152F). For some, treatment was successful in reducing or managing self-injury (three participants), such as the participant who ‘did 10 weeks of group DBT, was 18 months cutting free, the longest since I started’ (235F), and the participant who wrote, ‘I feel more confident and hurt myself less’ (74M).

In terms of thoughts of ending life, participants wrote frankly of the literal way that treatment and support was the reason ‘I am still alive’ (1F) and that ‘I would not be alive without it … as I’m quite sure I would have eventually succeeded in suicide without the help I’ve received over the years’ (435M). For other participants, suicide was no longer viewed as part of their future (seven participants):

The support I receive is very relevant … Due to my condition, I was in a position where I thought the idea of killing myself was better than carrying on. I see now that I had never learned how to ask for help. I’d spent my life camouflaging and thought I had to be able to do it all myself. I still find receiving the help a challenge but wholeheartedly know that it has been life-saving in the true sense of the word. I was at a rock-bottom with my self-esteem and sense of value. I am grateful for this relevant support as it is helping me through life-changes that I certainly could not do alone. I have faith that I can learn to value myself for who I am and maybe find a way of living peacefully. (178F)

Another outcome of positive experiences of treatment and support was of feeling ‘better able to function in your world’ (661M). With support, life had become ‘manageable’ (181F) and ‘help[ed] me cope better with life’ (143F). For some, these experiences made a ‘totally life changing difference to my life’ (620F). These experiences included an array of different capabilities, including relationships and everyday social interactions (15 participants):

I have been able to reduce the time that I spend feeling depressed and have been able to get into a healthy relationship. (582F)

In contrast to the aforementioned experiences of social isolation and exclusion from the community, participants wrote of how support had opened up their access (two participants):

To allow me to access the community when I was ill with mental health and therefore couldn’t go out alone and also attend activities and events that the people behind these events didn’t believe I could attend myself. (35F)

Participants also wrote of how not feeling alone (10 participants) made them feel ‘safe’ (28F) and provided them with ‘emotional reassurance’ (7F). One woman wrote of how the support alleviated pressure and ‘gives her the chance to step outside having to be the good mother and the good daughter and the good wife and just be me’ (175F). Participants wrote of better understanding themselves (nine participants) with improved ‘confidence’ (432M), ‘self-awareness’ (54F) and the ability to value themselves more: ‘my treatment has given me more compassion for myself as a human being’ (432M). Participants also described how support opened up employment and education prospects (11 participants): ‘when people understood my support needs it enabled me to survive, to work and to have good mental health’ (47M).

In stark contrast to the negative experiences described previously, these positive experiences reported a connection to others, acceptance, independence, autonomy and self-understanding (to name a few) and of suicide no longer being an option.

## Discussion

This study is the first to explore autistic adults’ experiences of treatment and support for mental health problems and suicidality. Previous research has shown that autistic adults are at high risk of experiencing mental health problems (Lever and Geurts, 2016), NSSI (Maddox et al., 2017), suicidality (Cassidy et al., 2014a) and unmet support needs (Cassidy et al., 2018a). Yet there is a lack of research, validated assessments and treatment approaches for mental health and suicidality in autistic adults (Cassidy and Rodgers, 2017) and knowledge of autism and mental health among professionals (Raja, 2014). This study thus aimed to identify barriers and enablers for autistic adults attempting to access support and treatment for these difficulties, to inform future research, policy and service planning for this group.

Participants in this study described being excluded from mental health services for a number of reasons. First, participants described a gap in available mental health services for autistic adults without co-occurring ID. Second, assumptions were made about autistic people being ‘high-functioning’ and perceived as coping when in fact they were struggling. Third, long waiting lists and lack of funding for support or treatment were described, even for autistic people experiencing suicidality. These results are consistent with previous research and clinical reports of lack of autism expertise in mental health and psychiatric settings (Raja, 2014) and no autism mental health pathway (Crane et al., 2018). A recent study on autism language-use from the perspective of the UK autism community, found the term ‘high-functioning’ can underestimate the problems an autistic person can face on a daily basis (Kenny et al., 2016). Bowker et al. (2011) also found that people with Asperger’s syndrome were less likely to receive treatment compared to other subtypes of autism, perhaps due to their less obvious symptoms or generally higher cognitive and intellectual abilities. This is particularly worrying given that up to 66% of adults newly diagnosed with Asperger’s syndrome have experienced suicidal ideation (Cassidy et al., 2014a), 72% of autistic adults score above the psychiatric cut off for suicide risk (Cassidy et al., 2018a) and autistic people without co-occurring ID are at the highest risk of dying by suicide than the general population (Hirvikoski et al., 2016).

Participants also described a lack of professionals’ understanding of autism, which contributed to barriers in accessing appropriate treatment and support. For example, many autistic people experience alexythymia – difficulty identifying and describing one’s own emotions (Bird et al., 2010), difficulties with social and communication skills (APA, 2013), and report ‘camouflaging’ their symptoms in order to fit in social situations (Hull et al., 2017; Lai et al., 2017; Rynkiewicz et al., 2016). Participants described how these difficulties led to challenges in recognising they needed help and successfully requesting help. In addition, autistic people not only have difficulty interpreting the behaviour of neurotypical adults (Cassidy et al., 2015; Cassidy et al., 2014b) but neurotypical adults may also have difficulty interpreting autistic peoples’ behaviour (Sheppard et al., 2016). This ‘double empathy problem’ could result in misunderstandings between autistic and non-autistic people (Milton, 2012). This may explain why participants described needing time to develop a rapport with their therapist, and of being misunderstood and feeling disregarded by professionals. Subsequently, participants described difficulty in coping with change (a key part of diagnostic criteria for autism, APA (2013), which meant they preferred consistent and long-term support and treatment. Hence, the participants in this study described how difficulties characteristic of autism, impacted their ability to recognise they need help, effectively request help, and benefit from help when it was finally acquired. Participants did not feel that it was their responsibility to provide training or educate professionals within services they came into contact with. Rather, professionals need autism training and appropriate tools to adapt to autistic peoples’ needs to enable them to effectively access and benefit from services.

Participants also described how their experiences of treatment and support impacted their well-being, of which mental health forms a key part (Burgess and Gutstein, 2007). Specifically, absence of, or inappropriate support or treatment not suited to their needs, was associated with feelings of disempowerment, perceived burdensomeness on family and friends, social exclusion and isolation, hopelessness and seeing suicide as an inevitability. In contrast, many participants described how appropriate tailored support and treatment empowered them, gave them autonomy, facilitated their inclusion in social networks and wider society and gave them hope for a future where suicide was no longer an option. Little is currently known about well-being in autism (Robeyns, 2016), but autonomy and a sense of belonging could be particularly important (Milton and Sims, 2016), similar to the general population (Pelton and Cassidy, 2017; Van Orden et al., 2010). Results from this study are consistent with these findings, and suggest that appropriate support could improve autistic adults’ well-being, of which autonomy and belonging are a crucial part.

Results have strong implications for services (such as the National Health Service (NHS) in the United Kingdom, referenced by many participants in this study). The majority of participants reported needing support with mental health, mentoring and social activities, but under half actually received this support. A previous study from this dataset showed that these unmet support needs significantly predicted increased risk of suicidality, after controlling for a number of known risk markers, such as employment and mental health problems (Cassidy et al., 2018a). Results from this study are consistent with these results and provide greater detail regarding enablers for autistic accessing services. Specifically, there needs to be greater availability of appropriate services for autistic peoples’ diverse needs, so these individuals do not ‘slip through the net’. Specific enablers identified include clinicians being both knowledgeable about mental health in autism and flexible in order to make reasonable adaptations to meet autistic peoples’ needs (Ghaziuddin et al., 2002). For example, autistic people may need support with emotional literacy prior to psychological therapies if they have difficulties identifying and describing their emotions. Given difficulties with communication, flexible thinking and adapting behaviour in many autistic people, psychological therapy is effective but takes longer and thus requires a higher number of sessions (Anderberg et al. 2017). Participants in this study described that it took much longer to establish a rapport with their therapist or support worker, but there were too few sessions, which caused much anxiety when they came to an end.

This study has a number of strengths. A key strength was the participatory approach used in the research, which ensured questions were important, relevant and clear to autistic people. The online recruitment method meant we were able to capture a large sample, and wide range and depth of experiences compared to interviews or focus groups, which are also less accessible to autistic people. However, respondents to the survey were autistic adults who were able to respond independently to an online survey, and therefore results are not generalisable to those with co-occurring intellectual disability. A majority of the sample comprised autistic women, opposite to the gender bias in the wider autistic population. A majority of participants had also experienced mental health treatment and may have responded to the survey as they felt it was important, and many therefore not be representative of those who did not respond. Diagnoses were all self-reported, however, previous research has shown good agreement between self- and clinician report (Daniels et al., 2011). The exploratory qualitative approach meant that it is not possible to draw conclusions on prevalence of these experiences in autism. However, given the openness of the questions, and the common themes among participants identified, gives strong evidence for shared experiences. Given the size of the dataset, it was only possible to display a snapshot of the main themes. The mean age of autism diagnosis was very broad (ranging from 2 to 59 years) and this study did not consider whether delayed diagnosis impacted the experiences of treatment and support. The lack of distinction between treatment and support in this study, owing to participants using the terms interchangeably, may have reduced the ability to make specific targeted improvements. Future quantitative research is needed to explore what types of adapted support and treatment could improve mental health outcomes and prevent suicide in autism, and whether the elements of well-being identified in the current and previous qualitative studies could inform a new measure of autistic well-being.

In summary, although participants reported experiences of being excluded from mental health services, with potentially tragic consequences for their well-being, there are also examples of participants benefitting from tailored support and treatment, which had a positive effect on their well-being. Given the potentially tragic consequences of failing to support autistic people and treat mental health problems and suicidality in this group, it is crucial that services are trained in autism and prepared to adapt to their needs. The participants’ experiences show that this is not only possible, but highly beneficial.

## Supplemental Material

AUT816053_Lay_Abstract – Supplemental material for ‘People like me don’t get support’: Autistic adults’ experiences of support and treatment for mental health difficulties, self-injury and suicidality Click here for additional data file. Supplemental material, AUT816053_Lay_Abstract for ‘People like me don’t get support’: Autistic adults’ experiences of support and treatment for mental health difficulties, self-injury and suicidality by Louise Camm-Crosbie, Louise Bradley, Rebecca Shaw, Simon Baron-Cohen and Sarah Cassidy in Autism

## Notes

1. We have opted to use identity first language throughout the article, as this is the most highly endorsed preference by the autistic community (Kenny et al., 2016). However, we acknowledge that a wide range of language is used to describe autism and many autistic people who took part in the study used person first language.

2. Random numbers in square brackets are used to distinguish participants anonymously, with ‘F’ denoting female and ‘M’ male participants. For example, (1F) refers to participant 1, female. Where more than one participant has endorsed a theme or phrase, frequency counts are given, for example (11 participants).

3. In this study, ‘learning disability’ used by participants predominantly in the United Kingdom is synonymous with ‘intellectual disability’ in the United States, meaning an intelligence quotient below 70.
