# Supplementary material for: ‘People like me don’t get support’: Autistic adults’ experiences of support and treatment for mental health difficulties, self-injury and suicidality
Source: Autism. 2018 Nov 29;23(6):1431–41. doi: 10.1177/1362361318816053 (PMC6625034; doi:10.1177/1362361318816053)
Supplement: AUT816053_Lay_Abstract – Supplemental material for ‘People like me don’t get support’: Autistic adults’ experiences of support and treatment for mental health difficulties, self-injury and suicidality [file AUT816053_Lay_Abstract.pdf]

Lay Abstract

autism

DOI: 10.1177/1362361318816053

‘People like me don’t get support’: Autistic adults’ experiences of support and treatment for mental health difficulties, self-injury and suicidality

Louise Camm-Crosbie, Louise Bradley, Rebecca Shaw, Simon Baron-Cohen[3,4] and Sarah Cassidy

Many autistic adults experience a mental health condition, or report feeling suicidal at some point in their lives. However, there are few assessment or treatment options for autistic people experiencing mental health problems, and none for those feeling suicidal. We also know very little about autistic peoples’ experiences of support and treatment for these difficulties. As we know so little, it is important that researchers work with the autistic community to make sure that research is useful to autistic people’s daily lives. We worked in partnership with a group of autistic adults who had experienced mental health problems or who had felt suicidal. Together, we designed an online survey to understand autistic peoples’ experiences of mental health problems, self-injury and thoughts of ending life, and what could help prevent these difficulties. One part of the survey asked autistic people to describe their experiences of treatment and support in relation to these difficulties. We found strong similarities in the experiences of autistic people. First, autistic people said it was very difficult for them to access support and treatment. Second, professionals were not trained to understand, support or treat autistic people experiencing mental health problems. Third, getting access to appropriate support and treatment was really important for autistic peoples’ wellbeing. This research will be used to make recommendations for services and practitioners to better support autistic adults experiencing mental health problems, self-injury and thoughts of ending life.
